# β-arrestin1-E2F1-ac axis regulates physiological apoptosis and cell cycle exit in cellular models of early postnatal cerebellum

**DOI:** 10.3389/fcell.2023.990711

**Published:** 2023-02-27

**Authors:** Luana Abballe, Vincenzo Alfano, Celeste Antonacci, Maria Giuseppina Cefalo, Antonella Cacchione, Giada Del Baldo, Marco Pezzullo, Agnese Po, Marta Moretti, Angela Mastronuzzi, Enrico De Smaele, Elisabetta Ferretti, Franco Locatelli, Evelina Miele

**Affiliations:** ^1^ Department of Pediatric Hematology/Oncology and Cellular and Gene Therapy, Bambino Gesù Children’s Hospital, Istituto di Ricovero e Cura a Carattere Scientifico, Rome, Italy; ^2^ Cancer Research Center of Lyon (CRCL), UMR Inserm U1052/CNRS 5286, Lyon, France; ^3^ Pathology Unit, Core Research Laboratories, Bambino Gesù Children’s Hospital, IRCCS, Rome, Italy; ^4^ Department of Molecular Medicine, Sapienza University, Rome, Italy; ^5^ Department of Experimental Medicine, Sapienza University, Rome, Italy; ^6^ Department of Gynecology/Obstetrics and Paediatrics, Sapienza University, Rome, Italy

**Keywords:** arrb1, E2F1, granule cell precursors (GCPs), neuronal stem cell (NSC), medulloblastoma (MB)

## Abstract

Development of the cerebellum is characterized by rapid proliferation of cerebellar granule cell precursors (GCPs) induced by paracrine stimulation of Sonic hedgehog (Shh) signaling from Purkinje cells, in the external granular layer (EGL). Then, granule cell precursors differentiate and migrate into the inner granular layer (IGL) of the cerebellum to form a terminally differentiated cell compartment. Aberrant activation of Sonic hedgehog signaling leads to granule cell precursors hyperproliferation and the onset of Sonic hedgehog medulloblastoma (MB), the most common embryonal brain tumor. β-arrestin1 (ARRB1) protein plays an important role downstream of Smoothened, a component of the Sonic hedgehog pathway. In the medulloblastoma context, β-arrestin1 is involved in a regulatory axis in association with the acetyltransferase P300, leading to the acetylated form of the transcription factor E2F1 (E2F1-ac) and redirecting its activity toward pro-apoptotic gene targets. This axis in the granule cell precursors physiological context has not been investigated yet. In this study, we demonstrate that β-arrestin1 has antiproliferative and pro-apoptotic functions in cerebellar development. β-arrestin1 silencing increases proliferation of Sonic hedgehog treated-cerebellar precursor cells while decreases the transcription of E2F1-ac pro-apoptotic targets genes, thus impairing apoptosis. Indeed, chromatin immunoprecipitation experiments show a direct interaction between β-arrestin1 and the promoter regions of the pro-apoptotic E2F1 target gene and *P27*, indicating the double role of β-arrestin1 in controlling apoptosis and cell cycle exit in a physiological context. Our data elucidate the role of β-arrestin1 in the early postnatal stages of cerebellar development, in those cell compartments that give rise to medulloblastoma. This series of experiments suggests that the physiological function of β-arrestin1 in neuronal progenitors is to directly control, cooperating with E2F1 acetylated form, transcription of pro-apoptotic genes.

## 1 Introduction

Medulloblastoma (MB) is the most common malignant brain tumor of childhood arising in the cerebellum. By large-scale omic studies conducted in the recent decades, four molecular subgroups have been universally recognized, termed WNT, Sonic hedgehog (SHH), Group 3 and Group 4. The different epigenetic and transcriptional profiles, as well as the specific genetic alterations, suggest that MB subgroups arise from distinct cell-of-origin or developmental lineages (Hovestadt et al., 2020).

In particular, two cell populations are known to give rise to SHH MB: granule cell precursors (GCPs) populations (ATOH1+) for the SHH MB subgroups and stem/progenitor cell populations for the MYCN- driven SHH MB (Hovestadt et al., 2020).

GCPs born in the rhombic lip (RL) of cerebellum at embryonic day 13 in mice migrate from the RL into the posterior dorsal region of RL, forming the external granular layer (EGL). After birth, for up to approximately 2 weeks, GCPs in the EGL continue to proliferate, in response to paracrine stimulation with Sonic Hedgehog (Shh) ligand from the underlying layer of Purkinje cells. Following the Shh proliferation-induced phase, GCPs differentiate, migrate to the internal granular layer (IGL) of the cerebellum to form a terminally differentiated granule layer, unresponsive to Shh stimuli (Ruiz i Altaba et al., 2002). This evidence suggests a regulatory mechanism that controls the response to Shh and cell cycle exit. Indeed, physiological widespread apoptosis characterizes GCPs when they exit the cell cycle during postnatal development (Ahlgren and Bronner-Fraser, 1999; Charrier et al., 2001).

The second MB cell-of-origin is the cerebellar neural stem cell (NSC) residing in the subventricular zone (Yang et al., 2008; Northcott et al., 2012), with a stem cell phenotype. NSCs can be derived from both cerebellum during development and in adulthood (Swartling et al., 2012). It is known that neuronal precursors death during differentiation is apoptotic in physiological development (Contestabile, 2002; Yeo and Gautier, 2004; Argenti et al., 2005; Allais et al., 2010) and that this process is regulated by signaling pathways rather than by the apoptotic machinery (Desagher et al., 2005). Recently, various mouse models (orthotopic, transgenic, and somatic gene transfer animals) have been used to demonstrate that stem/progenitor cells can be successfully transformed recapitulating the molecular and phenotypic characteristics of MYCN-driven SHH MB or MYCN- or MYC-driven Group 3 MB (Hovestadt et al., 2020).

β-arrestins proteins are the major transducers of G protein-coupled receptors (GPCRs) (Crépieux et al., 2017); they act by scaffolding proteins that can be activated independently, or in conjunction with G proteins, in both cytosol and nucleus. Moreover, in response to Shh stimuli, β-arrestin 1 (ARRB1) changes its subcellular localization and moves to a specialized structure required to Shh response, the primary cilium (Kovacs et al., 2008). In cerebellar NSCs, ARRB1 is epigenetically silenced to maintain stem cell features. The re-expression of ARRB1 enhances the cell cycle inhibitor P27 while inhibiting proliferative signaling, thus resulting in stem cell differentiation and growth arrest (Po et al., 2017). Moreover, in GCPs in which ARRB1 moved to the nucleus, it forms a complex with cofactors (P300 and CREB) increasing the transcription of *p27*, a differentiation marker of GCPs (Ma and Pei, 2007). Parathath and colleagues described a negative feedback mediated by Shh-stimulated ARRB1 driving to cell cycle exit through transcription enhance of P27 (Parathath et al., 2010).

E2F1 is a transcription factor implicated in the control of GCPs cell fate in the postnatal cerebellum (Wang et al., 2007). E2F1 has a central role in cell cycle progression interacting with retinoblastoma protein (pRB), but it is now clear that its function is not limited to cell cycle regulation, but also for tuning apoptosis, senescence and DNA-damage response (Denechaud et al., 2017). Depending on the interactors which it partners with, E2F1 can direct the GCPs towards cell proliferation and differentiation (RB/E2F1 complex) or towards apoptosis at the end of postnatal development of cerebellum (Suzuki et al., 2011). Moreover, an aberrant expression of E2F1 on GCPs has also been implicated in cerebellar neoplastic transformation (Suzuki et al., 2011) and its acetylation was increased when GCPs were stimulated with Shh (Miele et al., 2021).

Notably, in a recent study, we reported a new regulatory axis in which ARRB1 and E2F1 are critical for MB progression. Specifically, low expression of ARRB1 promotes tumor growth enhancing the E2F1 survival function, while high expression of ARRB1 triggers E2F1 acetylation switching E2F1 function from pro-survival into pro-apoptotic (Miele et al., 2021). However, the physiological mechanism of action of ARRB1 and E2F1 in regulating the two SHH MB cell-of-origin remains elusive.

In the present work, we identified a new crucial axis in two physiological neuronal cell models, in which ARRB1 works in partnership with acetylated E2F1 to guide the physiological apoptosis and growth arrest in GCPs and NSCs.

## 2 Materials and methods

### 2.1 Mice

Mice were purchased from Charles River Laboratories and maintained in the Animal Facility at Sapienza University of Rome. All procedures were performed in accordance with the Guidelines for Animal Care and Use of the National Institutes of Health with the approval of the Ethics Committee for Animal Experimentation (Prot. N 03/2013) of Sapienza University of Rome.

### 2.2 Murine cerebellum: Isolation and preparation

Cerebella were aseptically removed from CD1 mice at different stage of cerebellar development (day 2, 5, 7 and 15) and store at −80°. Murine cerebella were lysed in lysis buffer as described in “Western Blot analysis” section (see below). For immunohistochemical staining, cerebella from post-natal day 2, 5, 7, 10, and 15 were formalin-fixed and paraffin embedded (FFPE).

### 2.3 Murine cerebellar GCPs: Isolation, culture, and treatments

Cerebellar GCPs were prepared from 4-day-old CD1 mice according to established protocols (Wechsler-Reya and Scott, 1999; Argenti et al., 2005). Briefly, aseptically removed cerebella were cut into small pieces and incubated for 15 min at room temperature in D-PBS containing 0.1% trypsin, 0.2% EDTA, and 100 μg/ml DNase before being triturated with fire-polished Pasteur pipettes to obtain a single-cell suspension. After centrifugation, cells were resuspended in Neurobasal medium supplemented with B27, penicillin–streptomycin, and L-glutamine (2 mM) (Invitrogen) and plated at a density of 8 × 10^5^ cells/cm^2^ on tissue-culture dishes or eight-well Lab-Tek chamber slides (Permanox slide; Nunc, Naperville, IL) coated with 1 mg/ml poly-L-lys. GCPs were transfected with Arrb1 siRNA [ON-TARGETplus SMARTpool (L40976-00-005)] or pcDNA3 β-arrestin1 HA (gift from Robert Lefkowitz (Addgene plasmid # 14693) (Luttrell et al., 1999) using Lipofectamine 2000 3 h after plating (Invitrogen). GCPS were transfected at a rate of 15%–20%. Shh-stimulated GCPs were incubated with recombinant Shh (3 μg/ml) (R&D, Minneapolis, MN, United States) or BSA 0.1% (as control) for up to 48 h and cells were harvested for RNA/protein analysis.

### 2.4 Neural stem cells isolation, cultures and treatments

NSCs were isolated from mouse cerebella of 4-day-old CD1 mice, as previously described (Po et al., 2010) and were cultured in stem-cell medium (SCM) consisting of DMEM/F12 supplemented with 0.6% glucose, 25 mg/ml insulin, 60 mg/ml N-acetyl-L-cysteine, 2 mg/ml heparin, 20 ng/ml EGF, 20 ng/ml bFGF (Peprotech, Rocky Hill, NJ), 1X penicillin-streptomycin, and B27 supplement without vitamin A. For differentiation studies, NSCs were mechanically dissociated and plated into D-poly-lysine–coated dishes in differentiation medium (DFM) (DMEM/F12 with N2 supplement and 2 mg/ml heparin, 0.6% glucose, 60 mg/ml N-acetyl-L-cysteine, 1% FBS). Cells were harvested after 8, 16, and 24 h.

Amaxa nucleofector (Lonza) was used to transfect plasmids according to manufacturer’s procedure. pcDNA3 β-arrestin1 HA. Silencing of β-arrestin1 was performed using ON-TARGETplus SMARTpool (L40976-00-005) from Thermo Scientific. Transfection efficiency of ARRB1-HA overexpression experiments ranged between 80% and 90%. siGLO Red transfection control reagents (10 nM) (Dharmacon) were used to verify transfection efficiency that ranged between 75% and 85%.

### 2.5 RNA extraction and real-time PCR

Total RNA was purified and reverse transcribed as previously described (Spiombi et al., 2019). Quantitative RT-PCR (RT qPCR) analysis was performed using the ViiA 7Real-Time PCR System (Thermo Scientific), using best coverage TaqMan gene expression assays, specific for each analyzed mRNA.

Each amplification was performed in triplicate, and the average of the three threshold cycles was used to calculate the amount of transcripts (Thermo Scientific). Transcripts quantification was expressed in arbitrary units as the ratio of the sample quantity to the calibrator or to the mean values of control samples. All values were normalized to the 4 endogenous gene controls: *Gapdh*, *ß- Actin*, *ß2-microglobulin* and *Hprt.*

### 2.6 Western blot analysis

Murine cerebella and cells were lysed in Tris–HCl pH 7.6, 50 mM, deoxycholic acid sodium salt 0.5%, NaCl 140 mM, NP40 1%, EDTA 5 mM, NaF 100 mM, Na pyrophosphate 2 mM and protease inhibitors, while nuclear extraction was performed as already described (Po et al., 2017).

Lysates were separated on 8% or 6% acrylamide gel and immunoblotted using standard procedures. The following antibodies were used: anti-GLI1 (L42B10, Cell Signaling), anti-CASPASE-3 (D3R6Y, Cell Signaling), anti-β-ARRESTIN1 K-16 (sc-8182; Santa Cruz Biotechnology), anti-ACTIN I-19 (sc-1616; Santa Cruz Biotechnology), anti-E2F1 C-20 (sc-193; Santa Cruz Biotechnology), anti-E2F1 (acetyl K120/K125) (AP10555SU-N, Acris Antibodies); anti-ZIC1 (ab72694; Abcam); anti-PARP p85 Fragment (G7342; Promega), anti-SP1 1C6 (sc-420X; Santa Cruz Biotechnology), anti-PCNA (2586; Cell signaling), anti-H3 (ab 1971, Abcam), and anti-GAPDH (ab8245; Abcam). HRP-conjugated secondary antibodies (Santa Cruz Biotechnology) were used in combination with enhanced chemiluminescence (ECL Amersham).

### 2.7 Cell biology assays

Cell proliferation was evaluated by bromodeoxyuridine (BrdU) labeling assay (Roche) according to the manufacturer’s instructions. BrdU pulse was of 24 h and cells were counted in triplicate and the number of BrdU positive nuclei was annotated.

Apoptosis was detected by terminal deoxynucleotidyl transferase-mediated UTP nick end labeling (TUNEL) assay with the *In Situ* Cell Death Detection Kit Fluorescein (Cat. 702 No. 1684795, Roche Applied Sciences), according to the manufacturer’s instructions. Images were acquired with Carl Zeiss microscope (Axio Observer Z1) and AxioVision Digital Image Processing Software. Cells were counted in triplicate and the number of TUNEL-positive nuclei was annotated.

For overexpression experiments, a double-labeling assay for detecting apoptotic cells, using the terminal deoxy-nucleotidyl transferase (TdT)-mediated dUTP nick end-labeling (TUNEL) assay, and antigens of interest (pcDNA3 β-arrestin1 HA) using anti-HA (sc-7392 Santa Cruz), by immunofluorescence. Immunofluorescence staining was performed as already described (Po et al., 2017) using anti-β-ARRESTIN1 K-16 (sc-8182; Santa Cruz Biotechnology) for GCPs and anti-NANOG (REC-RCAB0002P-F; Cosmo Bio Co), anti-SOX2 (MAB4343; Millipore Billerica, MA), anti-NESTIN antibody (ab81462; Abcam), anti-GFAP monoclonal antibody (MAB360; Millipore), anti-PARVALBUMIN (P3088; Sigma); anti-S100 (S2644; Sigma); anti-β-III TUBULIN (MAB1637; Millipore) for NSCs.

To evaluate cell viability GCPs were plated at a density of 5 × 10^5^ cells/well in 96-well plates and were incubated with MTS solution (CellTiter 96^®^ AQueous One Solution Promega).

### 2.8 Chromatin immunoprecipitation

Chromatin immunoprecipitation (ChIP) analyses were performed on chromatin extracts according to manufacturer’s specifications of MAGnify Chromatin Immunoprecipitation System kit (Invitrogen). Sheared chromatin was immunoprecipitated with 5 µg of the following antibodies: anti-β-arrestin1 (Clone 10 cat. 610550 BD Biosciences) or normal mouse IgG, provided by the kit, was used as negative control. Eluted DNA was amplified by qPCR using EpiTect ChIP qPCR Assay (Qiagen) for the indicated genes (Mouse *Cdc25a, Trp73, Cdkn1b, Casp3, Casp7, Zeb1, Zeb2, Birc5, Vim* and *Fn1*). As control we used *Actin* and *Gapdh* gene. Data are presented as input percentage enrichment over background.

### 2.9 Immunohistochemistry (IHC)

For immunohistochemical staining, 5 μm FFPE sections were used. Mouse FFPE sections from different stage of cerebellum development (p2, p4, p7, p10, p15) were stained with the following primary antibodies: anti-CASPASE-3 (D3R6Y, Cell Signaling), anti-CDC25A (PA5-77902), anti-P27 (sc-1641; Santa Cruz Biotechnology) and then stained with hematoxylin and eosin (H&E).

CASPASE-3 and CDC25A indexes were generated based on the count of DAB positive cells on the total number of cells, quantified using the bioimage analysis software QuPath (Bankhead et al., 2017).

### 2.10 Statistical analysis

Statistical Analysis was performed using Prism software Version 6.0 (GraphPad, United States). Statistical differences were analysed by Mann–Whitney *U* test for non-parametric values and *p*-values lower than 0.05 were considered statistically significant. Results are expressed as means ± S.D.

## 3 Results

### 3.1 ARRB1 controls apoptosis and cell proliferation in granule cell progenitors (GCPs)

**FIGURE 1:**
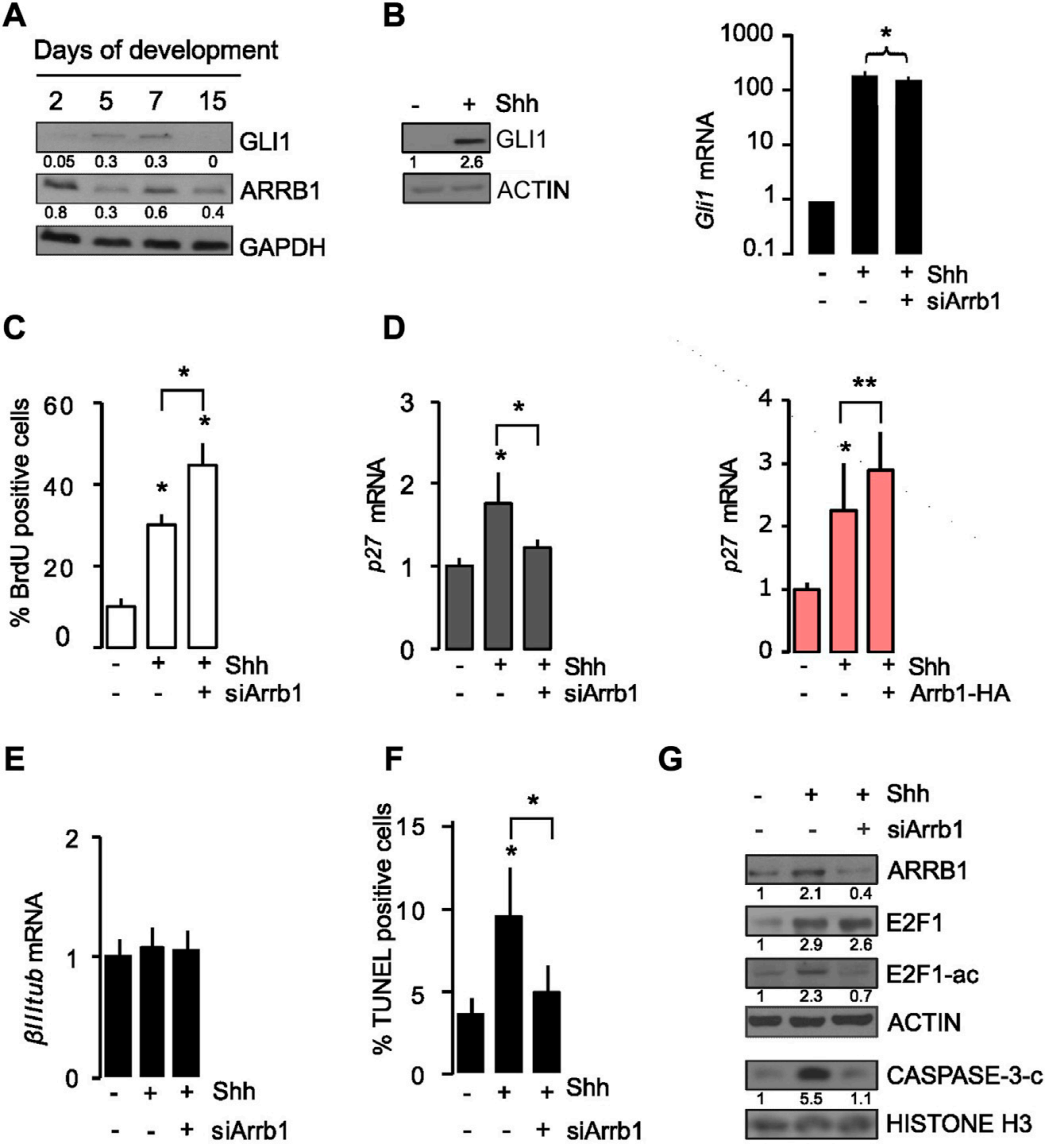
ARRB1 controls cell proliferation and apoptosis in granule cell progenitors (GCPs) *via* E2F1 acetylation. **(A)** Murine cerebellum Western blot. Representative images of endogenous GLI1 and ARRB1 in murine cerebella at different days (2, 5, 7, 15) of cerebellar development. GAPDH: loading control. **(B–G)** GCPs experiments. Shh was added to cultures of murine cerebellar GCPs that had or had not undergone siRNA-mediated silencing of ARRB1 (siArrb1). After 48 h of Shh stimulation, GCPs were assayed for: **(B)** *Gli1* mRNA and protein levels (as a read-out of Shh signaling activity), and ARRB1 protein level; ACTIN as loading control; **(C)** proliferation reflected by bromodeoxyuridine (BrdU) uptake; **(D)** left: *p27* mRNA levels (**(D)** right and Supplementary Figure S4B: Overexpression data are consistent); **(E)** differentiation reflected by *β III tubulin* mRNA levels; **(F)** apoptosis evaluated by TUNEL assay, and **(G)** ARRB1, E2F1, E2F1-ac, cleaved CASPASE-3 protein expression levels. ACTIN and HISTONE H3: loading controls. For Western blot, densitometry values are shown below the blots and densitometric graphs are presented in Supplementary Figure S2. Data represent means ± S.D., from at least three independent experiments; *p < 0.05; **p < 0.01; ***p < 0.001.

We wanted to elucidate the function of ARRB1 in the early postnatal stages of cerebellar development, in which GCPs proliferation, differentiation, and death are coordinated by Shh signaling (Wechsler-Reya and Scott, 1999). As expected, between 4 and 7 days after birth, undifferentiated GCPs are in proliferating stage under the effect of an active Hh signaling, whose activation is detectable by GLI1 protein expression levels. Subsequently, between 7 and 21 days after birth, GCPs progressively exit cell cycle, demonstrated by increased level of P27 (Supplementary Figure S1), and differentiate into mature granule cells (no detectable level of GLI1) [Figure 1 and (Ferretti et al., 2008)]. We observed detectable protein levels of ARRB1 at different stage of cerebellar development (2-, 5-, 7-, 15 post-natal day old mouse cerebella), under its physiological regulator Shh (Figure 1A; Supplementary Figure S2A). We observed a co-expression of GLI1 (as a readout of Shh signaling activation) and ARRB1 between 5 and 7 days of cerebellum development (Figure 1A). As already described, GLI1 regulates the shuttling of ARRB1 into the nucleus but not its transcription since ARRB1 is not a direct target of Gli1. For this reason, a concomitant increase in the two molecules is not appreciated. ARRB1 appeared to primarily function as a nuclear messenger for GCPs, likely providing scaffolds that regulate the localization and concentration of specific transcription factors at target gene promoters (Ma and Pei, 2007; Parathath et al., 2010).

Cerebellar GCPs were isolated according to procedures reported in the material and method section and we confirmed that GCPs expressed specific lineage markers such as ZIC1 and MATH1 (Supplementary Figures S2B, S3A). Based on previous findings (Parathath et al., 2010), exogenous Shh stimulation of cerebellar GCPs from post-natal day 4 mice significantly increased their GLI1 (both mRNA and protein levels) (Figure 1B; Supplementary Figure S2C). Interestingly, when such experiment was repeated on GCPs subjected to siRNA-mediated silencing of ARRB1 (si-Arrb1), the Shh-induced increase in proliferation was even more substantial reported as a percentage of BrdU positive cells (Figure 1C), while no difference was observed in terms of cell viability (Supplementary Figure S4A).

ARRB1 is known to interact with CREB and with the histone acetyltransferase P300 on the promoter of *P27*, enhancing its expression by acetylating histones H3 and H4 (Kang et al., 2005; Parathath et al., 2010). Our findings confirmed that the presence of ARRB1 in GCPs serves to terminate Shh-induced proliferation by increasing *P27*expression (Figure 1D). These findings are consistent with previous reports of a negative feedback loop, whereby mitogenic Shh signaling causes nuclear accumulation in cerebellar GCPs of the cyclin-dependent kinase inhibitor P27, which ultimately induces their cell-cycle exit (Parathath et al., 2010). In contrast, neither Shh stimulation nor ARRB1 depletion had any effect on GCPs differentiation, as reflected by *β* III TUBULIN (β*IIItub*) (Figure 1E) and ZIC1 (Supplementary Figures S2B, S3A).

Further on, considering Shh the major regulator of ARRB1 function in GCPs we examined cell apoptosis as it is a physiological process that plays fundamental roles in normal cerebellar development (Yeo and Gautier, 2004; Argenti et al., 2005). As shown in Figure 1F, Supplementary Figure S4B, Shh stimulation increased apoptosis of GCPs, and ARRB1 appears to be a key player in this effect given the significantly blunted apoptotic response observed in ARRB1-depleted cells and increased when ARRB1 is overexpressed. Accordingly, after Shh stimulation, GCPs increased the apoptotic PARP-C expression in the nucleus, together with ARRB1, while PARP-C protein decreased after si-Arrb1 (Supplementary Figures S2D, S4C). Moreover, no modulation of cell cycle associated protein (PCNA) was observed in siRNA experiments (Supplementary Figures S2D, S4C).

Cerebellar GCPs apoptosis is also reportedly dependent on expression of the transcription factor E2F1 (O’Hare et al., 2000). However, in normal and neoplastic lung cells, ARRB1 binds E2F-responsive promoters of genes that promote cell proliferation and survival (Dasgupta et al., 2011). The Janus-like behavior of E2F1 is controlled by its acetylation status (Pediconi et al., 2003). Acetylation of E2F1 “shifts its attention” from target genes that promote cell cycle progression (e.g., Cell division cycle 25a - *Cdc25a*, Thymidylate synthetase -*Tyms*, and Baculoviral IAP repeat–containing 5- *Birc5*) (Dasgupta et al., 2006; 2011) or epithelial mesenchymal transition (EMT) (e.g., Zinc Finger E-Box Binding Homeobox 1-*Zeb1*, Zinc Finger E-Box Binding Homeobox 2-*Zeb2*, Vimentin and Fibronectin 1-*Fn1* (Pillai et al., 2015) to those that are pro-apoptotic, including Transformation-related protein 73 (*Trp73*), Caspase 3 (*Casp3*) and Caspase 7 (*Casp7*) (Pediconi et al., 2003; Ianari et al., 2009). Therefore, the role of ARRB1 in GCPs apoptosis might conceivably be related to its effects on E2F1 acetylation.

Previous evidence (Miele et al., 2021) showed that E2F1 and ARRB1 co-immunoprecipitated in GCPs and the acetylation of E2F1 (E2F1-ac) are induced by overexpression of ARRB1. Based on these data, we demonstrated that silencing of ARRB1 had no effect on the abundance of E2F1 protein, but it appreciably diminished levels of the acetylated form and cleaved form of CASPASE 3 (CASP-3-C), one pro-apoptotic target of E2F1-ac (Figure 1G; Supplementary Figure S2E).

Collectively, these findings highlight two critical roles for ARRB1 in physiological neuronal cell models: induction of GCPs apoptosis by the acetylation of E2F1 and termination of cell cycle progression by enhancing P27 expression.

### 3.2 ARRB1 controls GCPs apoptosis *via* acetylated E2F1 pro-apoptotic targets

We evaluated the target genes regulated by ARRB1 in Shh treated GCPs. In particular, we evaluated whether ARRB1 mediates the induction of genes known to be targets of E2F1-ac: i) Pro-apoptotic genes: *Trp73, Casp3* and *Casp7*; ii) Pro survival/proliferative genes: *Cdc25a, Birc5*, and *Tyms*; iii) Genes involved in epithelial mesenchymal transition: *Zeb1, Zeb2, Vimentin* and *Fn1*.

**FIGURE 2:**
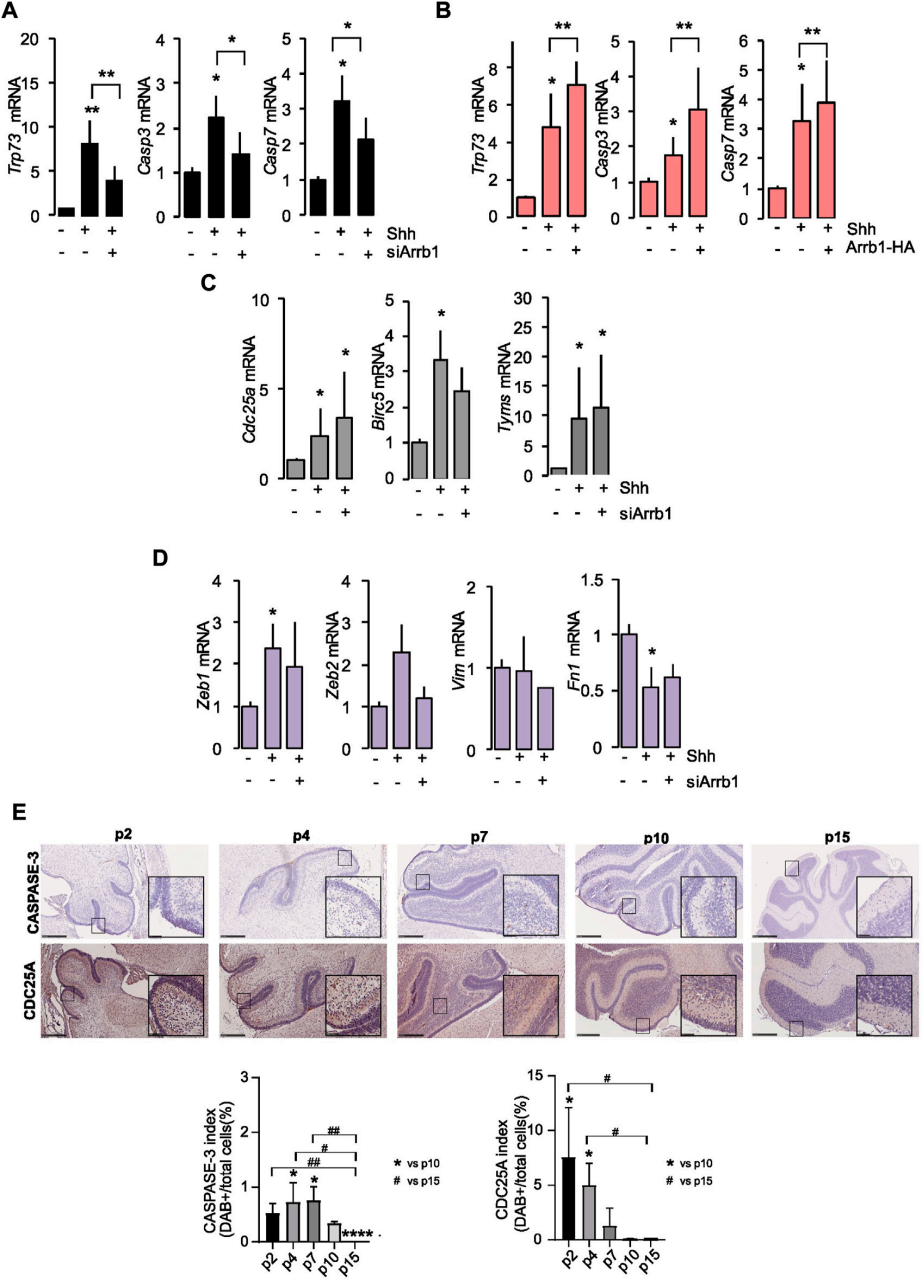
E2F1-ac targets’ transcripts after ARRB1 modulation. **(A,B)** Expression of pro-apoptotic E2F1-ac target genes (*Trp73, Casp3* and *Casp7*) in GCPs that had or had not undergone siArrb1 (black) or over-expression of Arrb1 (Arrb1-HA) (orange). **(C,D)** Expression of proliferative (grey) and epithelial mesenchymal transition (violet) E2f1-ac target genes (*Cdc25a, Birc5, Tyms, Zeb1, Zeb2, Vim* and *Fn1*) in GCPs that had or had not undergone siArrb1. Data represent means ± S.D., from at least three independent experiments; *p < 0.05; **p < 0.01; ***p < 0.001. **(E)** IHC staining for ARRB1 and P27 in representative mouse cerebellum sections at several differentiation stages (p2, p4, p7, p10, p15) (upper panels). Magnification ×10; insets ×40. Scale bar, 250 µm. CASPASE-3 and CDC25A indexes (expressed as percentage) calculated as DAB positive cells of total number of cells (bottom panels). Data represent means ± S.D., from at least three independent experiments; *p < 0.05; **p < 0.01; ***p < 0.001; ****p < 0.001 vs. p10. ^#^ *p* < 0.05; ^##^ *p* < 0.01 vs. p15.

We observed by RT qPCR that among the E2F1-ac targets induced by Shh, ARRB1 regulated only the pro-apoptotic genes *Caspase 3, Caspase 7, Trp73*, as shown by both ARRB1 silencing and overexpression experiments (Figures 2A, B).

On the other hand, the proliferative (*Cdc25a, Birc5*, and *Tyms*) and the EMT (*Zeb1* and *Fn1*) E2F1-ac target genes were controlled by Shh without requiring the presence of ARRB1 (Figures 2C, D). We did not observe a significant modulation of the other EMT genes (*Zeb2* and *Vim*) neither under Shh stimulation, nor after ARRB1 silencing (Figure 2D).

To support our mRNA data, we evaluated by IHC two of the E2F1-ac target proteins (CASPASE 3 and CDC25A) during mouse cerebellum development (from p2 to p15). As shown in Figure 2E the expression level the pro-apoptotic CASPASE 3 was expressed from p2 to p10, with a peak on p4/p7 in a context of Shh stimulation and ARRB1 expression (Supplementary Figure S2E). Instead, the proliferative CDC25A followed a significant negative trend during development, decreased from p2 to p10/p15 (Supplementary Figure S2E).

Collectively, these findings demonstrated the critical role of ARRB1 in normal cerebellar development in induction of GCPs apoptosis *via* E2F1-ac pro-apoptotic genes.

### 3.3 ARRB1-E2F1 complex direct regulates the expression of E2F1-ac pro-apoptotic target genes

**FIGURE 3:**
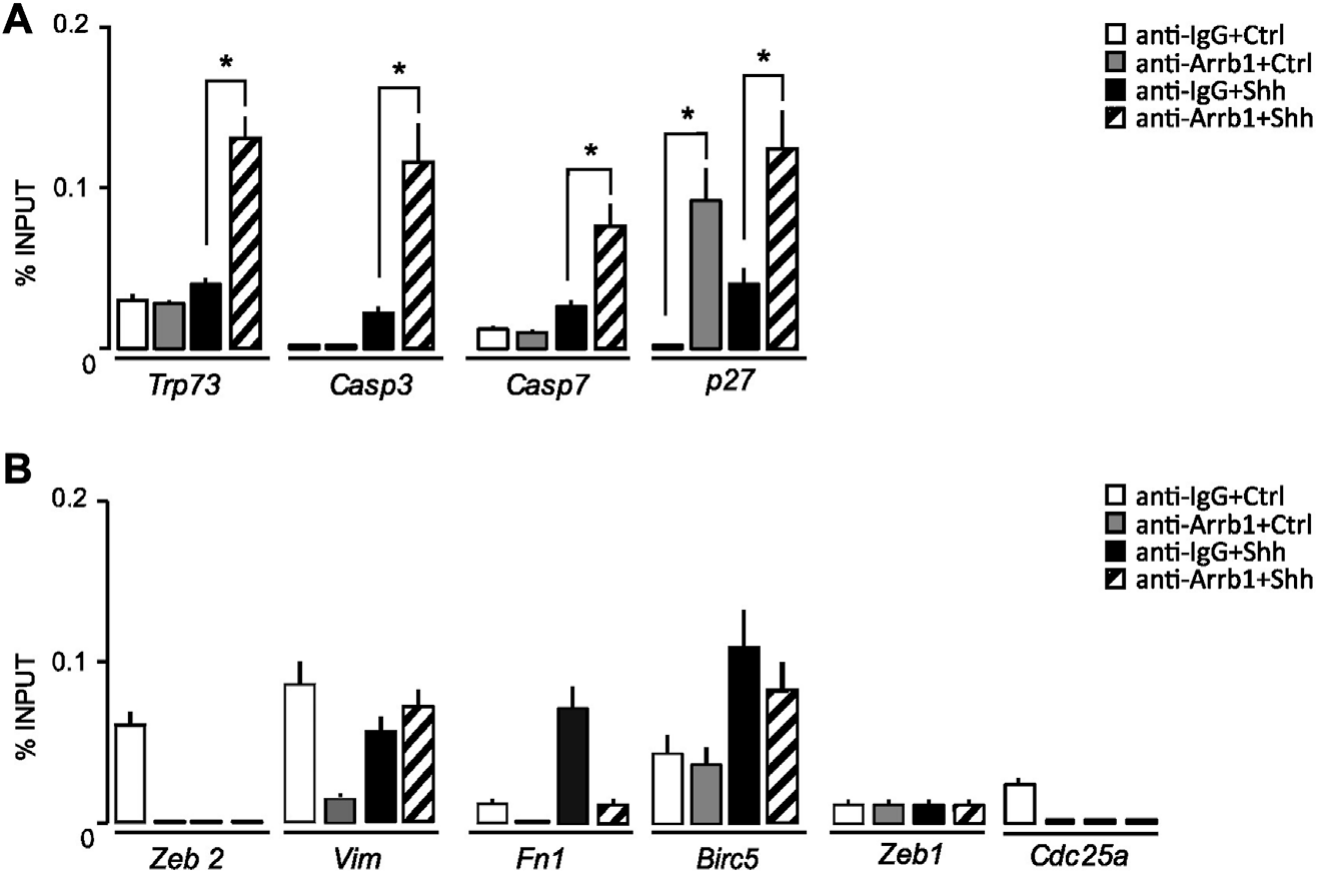
ARRB1-E2F1 complex direct regulates the expression of E2F1-ac pro-apoptotic targets. **(A,B)** qPCR-ChIP assay of ARRB1 in GCPs stimulated or not with Shh. Immunoprecipitation with IgG was performed as control. Eluted DNA was amplified by qPCR using primers specific for the regulatory region of the indicated genes. *Actin* and *Gapdh* (not shown) were used as endogenous non-enriched regions. qPCR data are presented as percentage of ChIP input controls. Data represent means ± S.D., from at least three independent experiments; *p < 0.05; **p < 0.01; ***p < 0.001.

Such ARRB1 function was validated by chromatin immunoprecipitation (ChIP) experiments. In Shh treated GCPs, ARRB1 mediates the binding of E2F1 to the promoter region of *Trp73, Casp3* and *Casp7* highlighting the role of ARRB1 in the regulation of apoptosis (Figure 3A); while it does not bind to the promoter of pro survival/proliferative genes as *Birc5* and *Cdc25a*, and to epithelial mesenchymal transition genes as *Zeb2, Vimentin, Fn1* and *Zeb1* (Figure 3B).

We found that ARRB1 also bound to the *Cdkn1b/p27* promoter, strengthening support for ARRB1’s role in GCPs growth arrest (Figure 3A).

Collectively, these findings demonstrated the direct controls of ARRB1-E2F1-ac complex on pro-apoptotic targets’ promoter regions.

### 3.4 ARRB1 controls apoptosis *via* E2F1-ac target in neural stem cells

**FIGURE 4:**
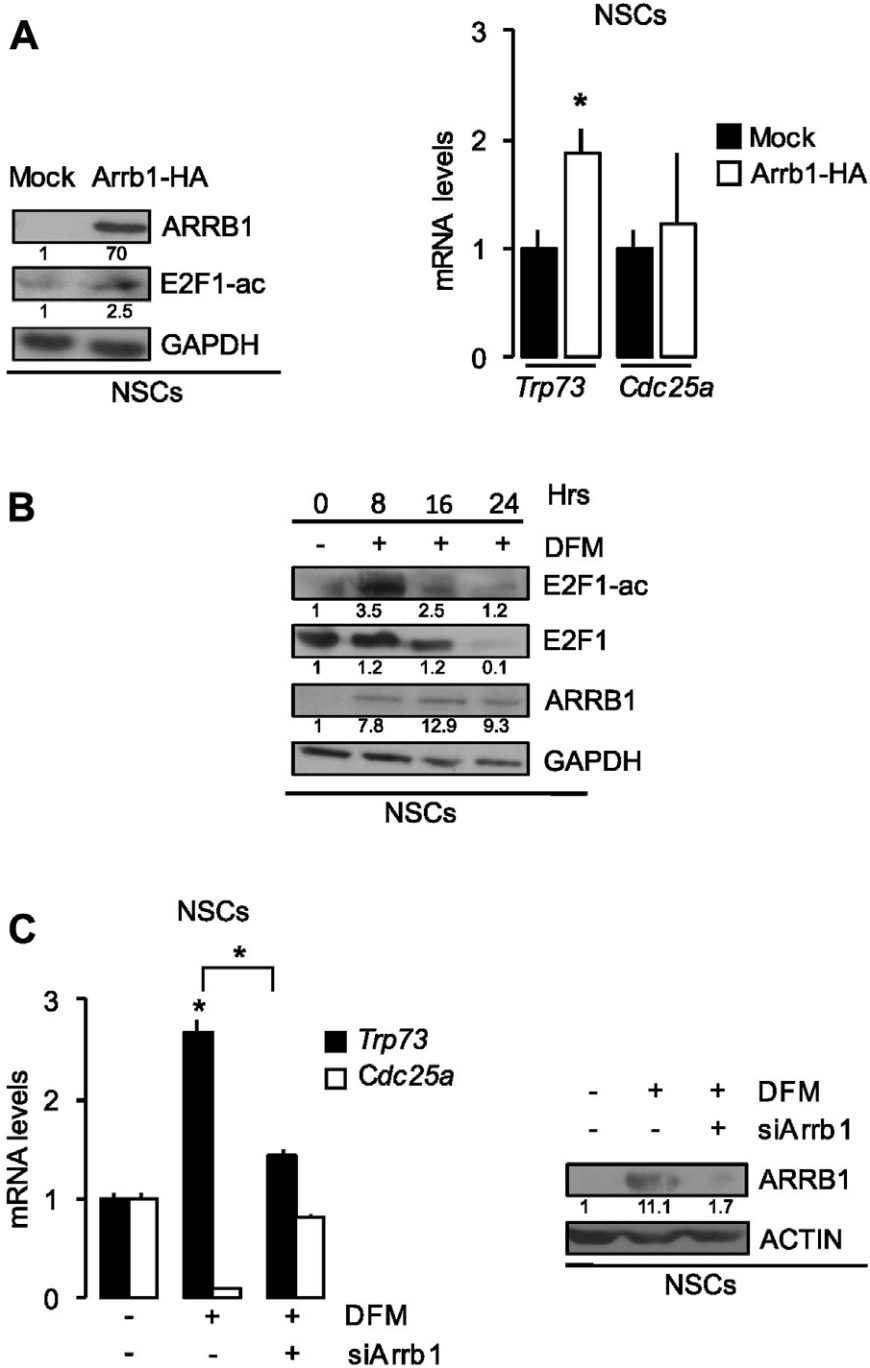
ARRB1 controls NSCs apoptosis *via* E2F1 acetylation. **(A)** Effects of Arrb1-HA overexpression and mock transfection (control) of NSCs on ARRB1 and E2F1-ac protein levels (Western blot assay-left) and *Trp73* and *Cdc25a* mRNA levels (right). *p < 0.05 vs. mock transfected cells (Mock). GAPDH: loading control for Western blot. **(B)** Western Blot analysis of endogenous ARRB1, E2F1, and E2F1-ac expression in NSCs cultured in SCM (0 h) and after 8–24 h culture in DFM. GAPDH: loading control. Effects of si-Arrb1 or scrambled control (siCtr) on **(C)** *Trp73* and *Cdc25a* mRNA levels (left) and ARRB1 protein levels (Western blot assay-right) in NSCs cultured in SCM (0 h) and after 18 h culture in DFM. ACTIN: loading control for Western blot. For Western blot, densitometry values are shown below the blots and densitometric graphs are presented in Supplementary Figure S2. *p < 0.05 vs. scrambled control (siCtr).

The early postnatal murine cerebellum contains multipotent NSCs [described by (Lee et al., 2005)] which can also give rise to SHH MB (Northcott et al., 2012). For this reason, we investigated the physiological roles of ARRB1 in these cells obtained from the cerebellum of 4 days old WT mice (Ferretti et al., 2008). NSCs growing in stem medium expressed very low levels of ARRB1 (Po et al., 2017) (Figure 4A; Supplementary Figure S2F), conversely they were positive for neuronal marker β-III TUBULIN (TUBB3) and stemness markers NANOG, NESTIN, SOX2, (Supplementary Figure S3B). On the other hand, ARRB1 overexpression is linked to a “differentiated neural phenotype” (Po et al., 2017), confirmed by the expression of differentiation markers [TUBB3, S100, PARVALBUMIN (PARV) and GFAP] (Supplementary Figure S3C).

Here, we demonstrated that ectopic expression of ARRB1 in NSCs increased E2F1 acetylation and enhanced transcription of pro-apoptotic E2F1-ac target genes (e.g., *Trp73*) but not those with proliferative effects (e.g., *Cdc25a*) (Figure 4A).

As shown in Figure 4B and Supplementary Figure S2G, in NSCs E2F1 is not acetylated in the absence of ARRB1, while in differentiating conditions, ARRB1 protein is expressed and increased E2F1 acetylation together with the expression of its target *Trp73* (Figure 4C; Supplementary Figure S2H). In line with these observations, ARRB1 depletion reduced *Trp73* transcription in differentiating NSCs but had no effect on *Cdc25a* transcription (Figure 4C).

Consistent with the role of ARRB1 observed in GCPs, in differentiating NSCs where ARRB1 is expressed and E2F1 is acetylated, ARRB1 protein is expressed and induced pro-apoptotic E2F1-ac target genes transcription.

**FIGURE 5:**
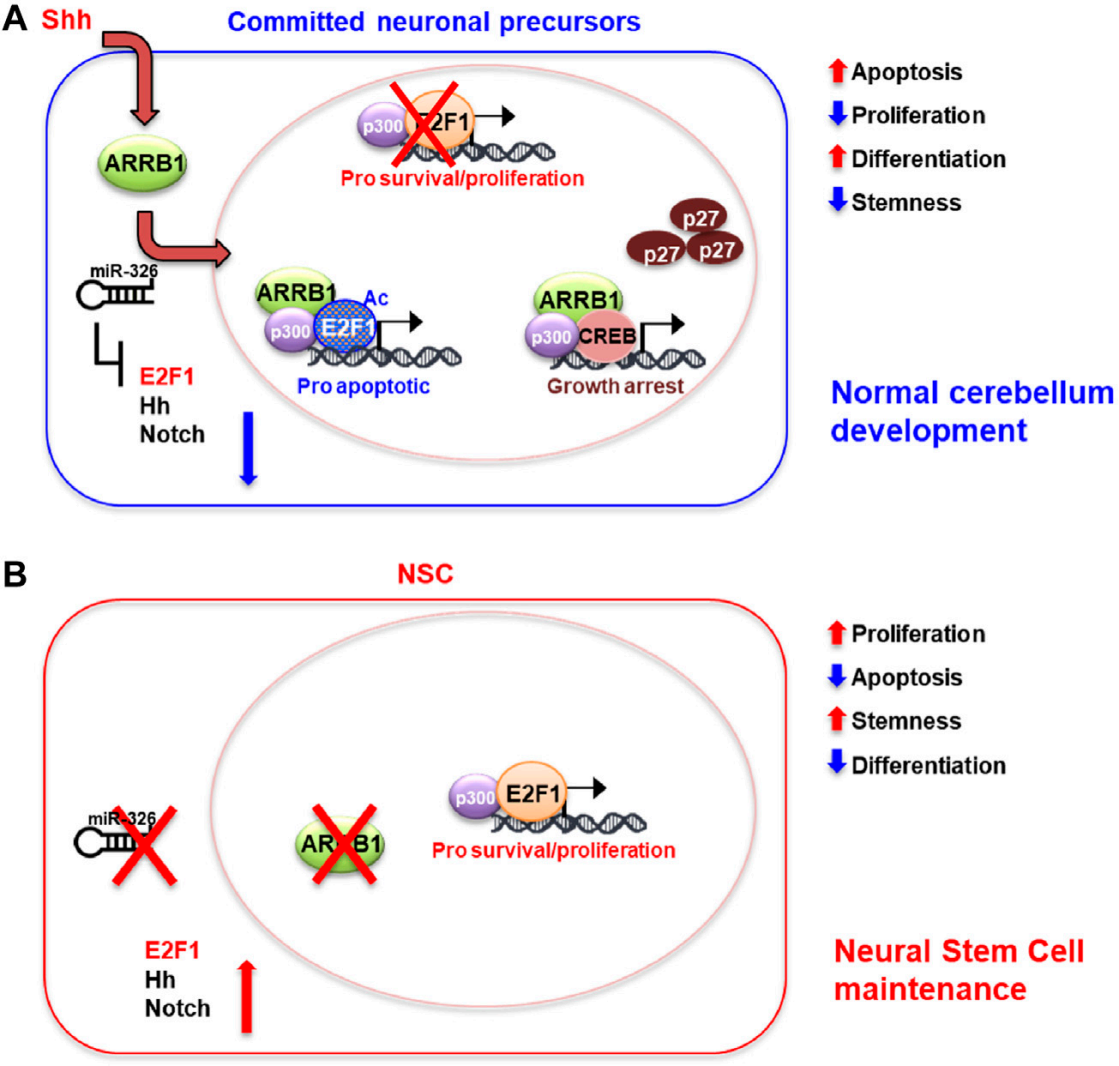
Schematic model of ARRB1/E2F1-ac functions in GCPs and NSCs. Overview of roles played by ARRB1/E2F1-ac in normal cerebellar development. **(A)**: Committed neuronal precursors (i.e., NSCs grown in DFM, GCPs). In our previous works, we identified miR-326 as a miRNA necessary for maturation of granule cell progenitors (GCPs) into mature granule cells (Ferretti et al., 2008). Moreover, this miRNA is integrated into the first intron of the *Arrb1* gene and shares the same regulatory regions as its host gene. miR-326 also contributes to ARRB1 functions by blunting proliferative signals mediated by E2F1, Hedgehog, and Notch, and by promoting cell differentiation (Ferretti et al., 2008; Kefas et al., 2009; Po et al., 2017; Miele et al., 2021). Committed neuronal precursors express ARRB1 and mir-326, which regulate their development at multiple levels. Shh signaling upregulates ARRB1 levels and promotes its translocation to the nucleus. There ARRB1, in complex with P300, induces acetylation of E2F1 (E2F1-ac), redirecting the transcription factors activity from survival/proliferative gene targets towards those that promote apoptosis (*Trp73, Caspases 3* and *7*). Interacting with CREB and P300, ARRB1 upregulates the expression and nuclear accumulation of P27, which eventually blocks cell cycle progression. miR-326 favors neuronal cell differentiations by inhibiting multiple survival/proliferative signaling: E2F1, Hedgehog (Hh) and Notch *via* direct binding of the 3′-UTRs of *E2f1, Smo, Gli2, Notch1* and *Notch2*. **(B)**: In NSCs, non-expression of ARRB1 and miR-326 promotes cell proliferation, survival, and stemness by favoring non-acetylated E2F1 activity and active Hedgehog (Hh) and Notch signaling.

The results in this physiological context allow us to propose a model where ARRB1 is involved in apoptosis and cell cycle exit in committed precursors to favor cell differentiation (Figure 5).

## 4 Discussion

In this study, we show the key roles of ARRB1/E2F1-ac axis *in vitro* experiments using two cellular populations that give rise to SHH MB: cerebellar GCPs and NSCs, useful models to mimic the normal cerebellar environment. Notably, the dysregulation of this process is a major promoter of tumor cell growth in MB (Miele et al., 2021).

The developing cerebellum needs a proper and timely balance between cell proliferation, survival, differentiation and apoptosis, this latter is a hallmark feature of CNS development (Yeo and Gautier, 2004).

ARRB1 is known to regulate multiple intracellular signaling pathways, many of which are involved in “life-or-death” balance in the cell (Lefkowitz and Shenoy, 2005; Gurevich and Gurevich, 2006). ARRB1 was described as a scaffolding protein that shuttles between the cytoplasm and the nucleus, where it interacts with CREB and the acetyltransferase P300 on the promoters of its target genes (Kang et al., 2005; Parathath et al., 2010). The functional consequences of ARRB1’s nuclear activity are less clear than the cytoplasmic ones, and many appear to be cell type- and/or context-specific. ARRB1 transcriptionally regulates genes involved in cell-cycle arrest/differentiation (*p27, c-fos*), those involved in proliferation/survival by recruiting E2F1 (*cAbl, Bcr/Abl*), *Cdc25A, Tyms,* and *Birc5* (Dasgupta et al., 2011; Qin et al., 2014) as well as those controlling apoptosis (*Trp73, Caspase 3 and Caspase 7*), mediated by the binding with the E2F1 acetylated form (Miele et al., 2021).

To evaluate ARRB1’s role within physiological cerebellar models, we analyzed its temporal expression at different stages of cerebellum development (from 2 to 15 days), and we carried out experiments modulating its levels in two cerebellar cell models, GCPs and NSCs.

In committed neuronal precursors (GCPs), we carried out experiments modulating β-arrestin-1 levels after Shh and chromatin immunoprecipitation experiments in GCPs to assess its role in this context. We found that ARRB1, in GCPs, as shown in Figure 5, acts in concert with its molecular partners (E2F1-ac) to ensure normal cerebellar development. In the present work we demonstrated that ARRB1 exerts its physiological nuclear functions at two levels: a) by the activation of the cell cycle exit *via* P27 (Figures 1–3), and b) by enhancing the acetylation of E2F1, redirecting its functions to non-proliferative ones. Indeed, ARRB1 promotes the acetylation of E2F1 under Shh stimuli and induce apoptosis *via* the pro-apoptotic targets of E2F1-acetylated (*Trp73, Caspases 3* and *7*) (Figures 2, 3). Our findings are consistent with reports of the diffuse, physiological GCPs apoptosis (Ahlgren and Bronner-Fraser, 1999; Charrier et al., 2001).

In the other cell model analyzed, neural stem cells (NSCs), ARRB1 is epigenetically suppressed, such as other developmental genes, during the expansion phase of the cerebellar pool ((Burgold et al., 2008) and this report), to favor the proliferation and survival. Later, when the pool of NSCs has expanded, ARRB1 expression is reactivated to terminate the proliferative phase and allow the precursors to undergo differentiation or apoptotic elimination (Wechsler-Reya and Scott, 1999; Po et al., 2017). Coherently, our result demonstrated that the ectopic expression of ARRB1 in NSCs induced the expression of acetylated E2F1 and its target *Trp73* (Figure 4A). Moreover, the endogenous expression of the ARRB1, under differentiation conditions, regulated the transcription of pro-apoptotic genes such as *Trp73* but not that of the proliferative genes (*Cdc25a*) (Figures 4B, C).

MiR-326 also contributes to this process by blunting proliferative signals mediated by E2F1, Hedgehog, and Notch, and by promoting cell differentiation as already reported (Ferretti et al., 2008; Kefas et al., 2009; Po et al., 2017; Miele et al., 2021).

Altogether, our results provide a significant contribution to elucidate a molecular mechanism through which ARRB1 mediates apoptosis and cell cycle exit in the two cells of origin of SHH-MB: cerebellar granule neuron precursors and neural stem cells.

## Data Availability

The original contributions presented in the study are included in the article/Supplementary Material, further inquiries can be directed to the corresponding authors.

## Supplementary material

SUPPLEMENTARY FIGURE S1 Expression levels of P27 during mouse cerebellum development. IHC staining for P27 in representative mouse cerebellum sections at several differentiation stages (p2, p4, p7, p10, p15). Magnification 10X; insets 40X. Scale bar, 250 µm.

SUPPLEMENTARY FIGURE S2 Densitometric analysis. **(A-H)** Densitometric graphs of the western blots presented in the manuscript. Data represent means ± S.D., from at least three independent experiments.

SUPPLEMENTARY FIGURE S3 Characterization of cellular models. **(A)** Left: CT values (by RT qPCR) of GCPs markers (*Math1* and *Zic1*), and *β-2-microglubulin* (as housekeeping gene), evaluated in silencing experiment on GCPs. Right: Western Blot analysis of endogenous expression of ZIC1, a GCPs marker, in GCPs that had or had not undergone siArrb1. GAPDH: loading control. **(B)** Representative image of immunofluorescence staining of NSCs for stemness/neuronal markers (NANOG, NESTIN, SOX2 and TUBB3). Nuclei are counterstained with Hoechst. Scale bar: 10 μm. **(C)** Representative image of immunofluorescence staining of differentiated NSCs for differentiation markers (TUBB3, S100, PARV and GFAP). Nuclei are counterstained with Hoechst. Scale bar: 10 μm. For western blot, densitometry values are shown below the blots and densitometric graphs are presented in Supplementary Figure S2.

SUPPLEMENTARY FIGURE S4 ARRB1 overexpression increases apoptosis in GCPs. **(A)** Evaluation of cell viability, measured with MTS assay, of GCPs treated for 48 h with Shh had or had not undergone siRNA-mediated silencing of ARRB1 (siArrb1). Statistical differences (versus untreated cells, ns = not significant) were evaluated by One-Way ANOVA. Data represent means ± S.D., from at least three independent experiments. **(B)** Apoptosis evaluated by a double-labeling assay for detecting apoptotic cells (TUNEL assay), and Arrb1 overexpressed (pcDNA3 β-arrestin1 HA) using anti-HA antibody, by immunofluorescence. Data represent means ± S.D., from at least three independent experiments; *p < 0.05; **p < 0.01; ***p < 0.001. **(C)** Nuclear subcellular localization of endogenous ARRB1, PARP-C and PCNA by Western blot (SP1 was used as loading controls and markers for purity of nuclear fraction). For western blot, densitometry values are shown below the blots and densitometric graphs are presented in Supplementary Figure S2.
