# Supplementary figures and images for: β-arrestin1-E2F1-ac axis regulates physiological apoptosis and cell cycle exit in cellular models of early postnatal cerebellum
Source: Front Cell Dev Biol. 2023 Feb 27;11:990711. doi: 10.3389/fcell.2023.990711 (PMC10010392; doi:10.3389/fcell.2023.990711)

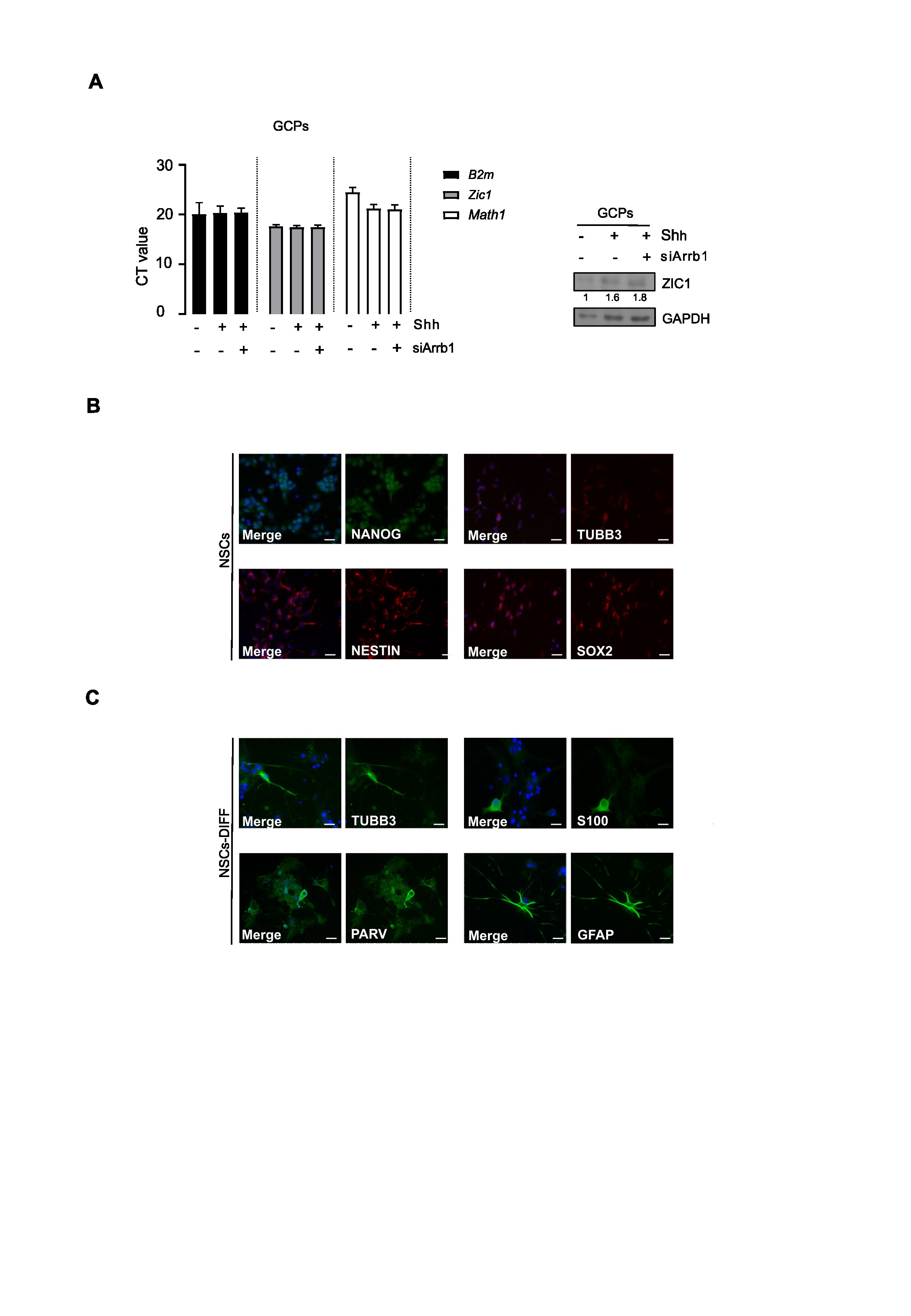

Supplement: Supplementary file 1 [file Image3.JPEG]

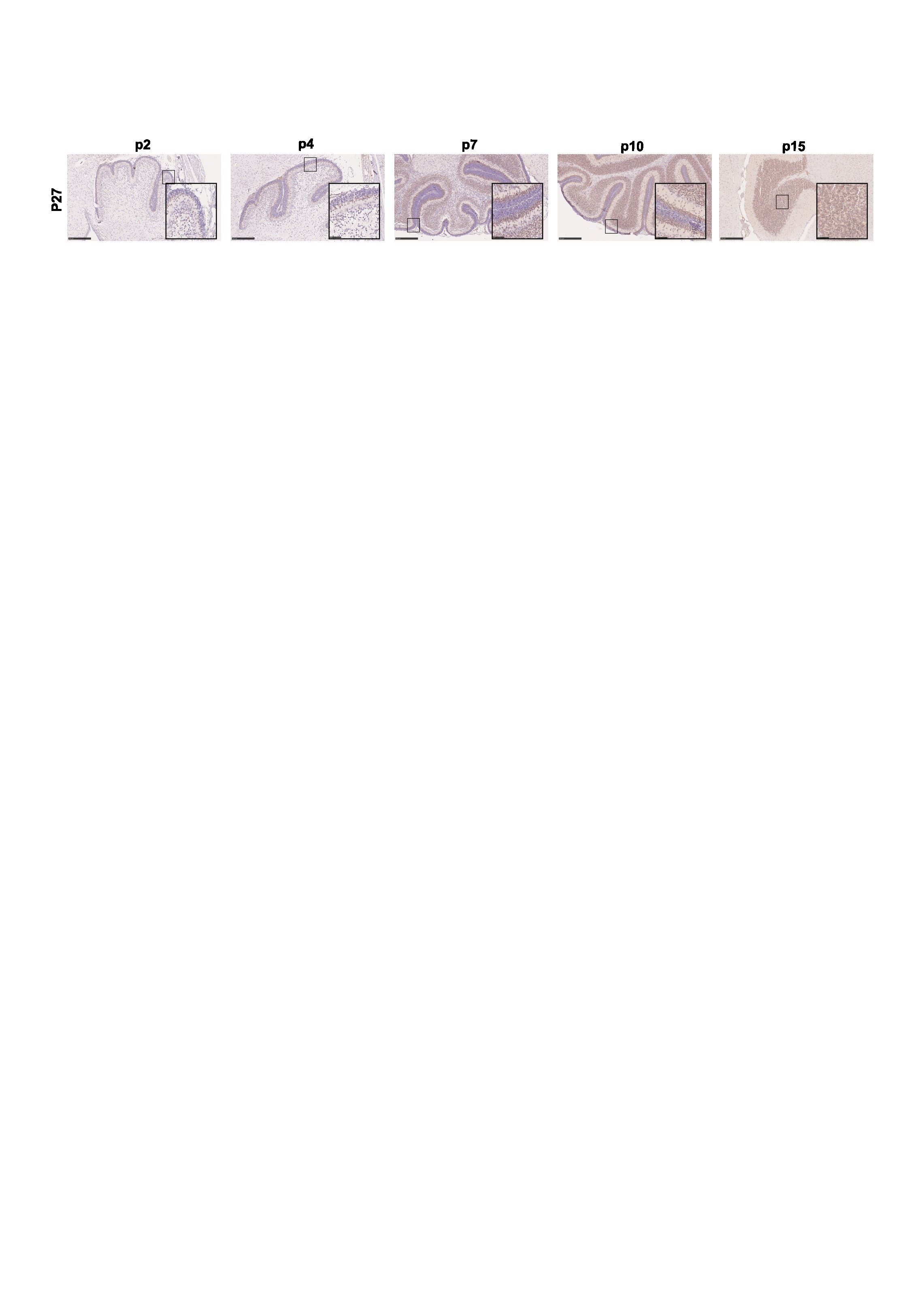

Supplement: Supplementary file 2 [file Image1.JPEG]

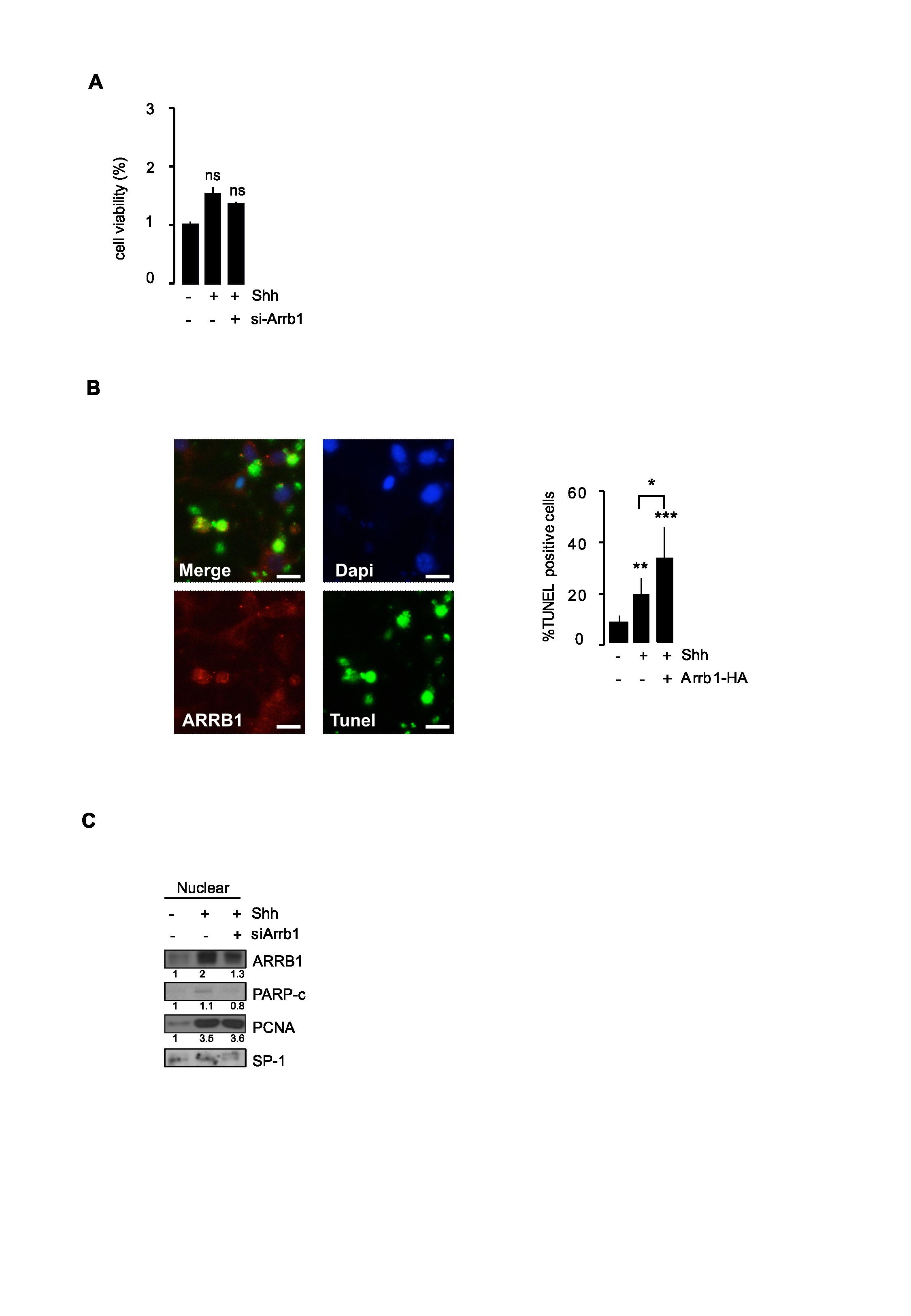

Supplement: Supplementary file 3 [file Image4.JPEG]

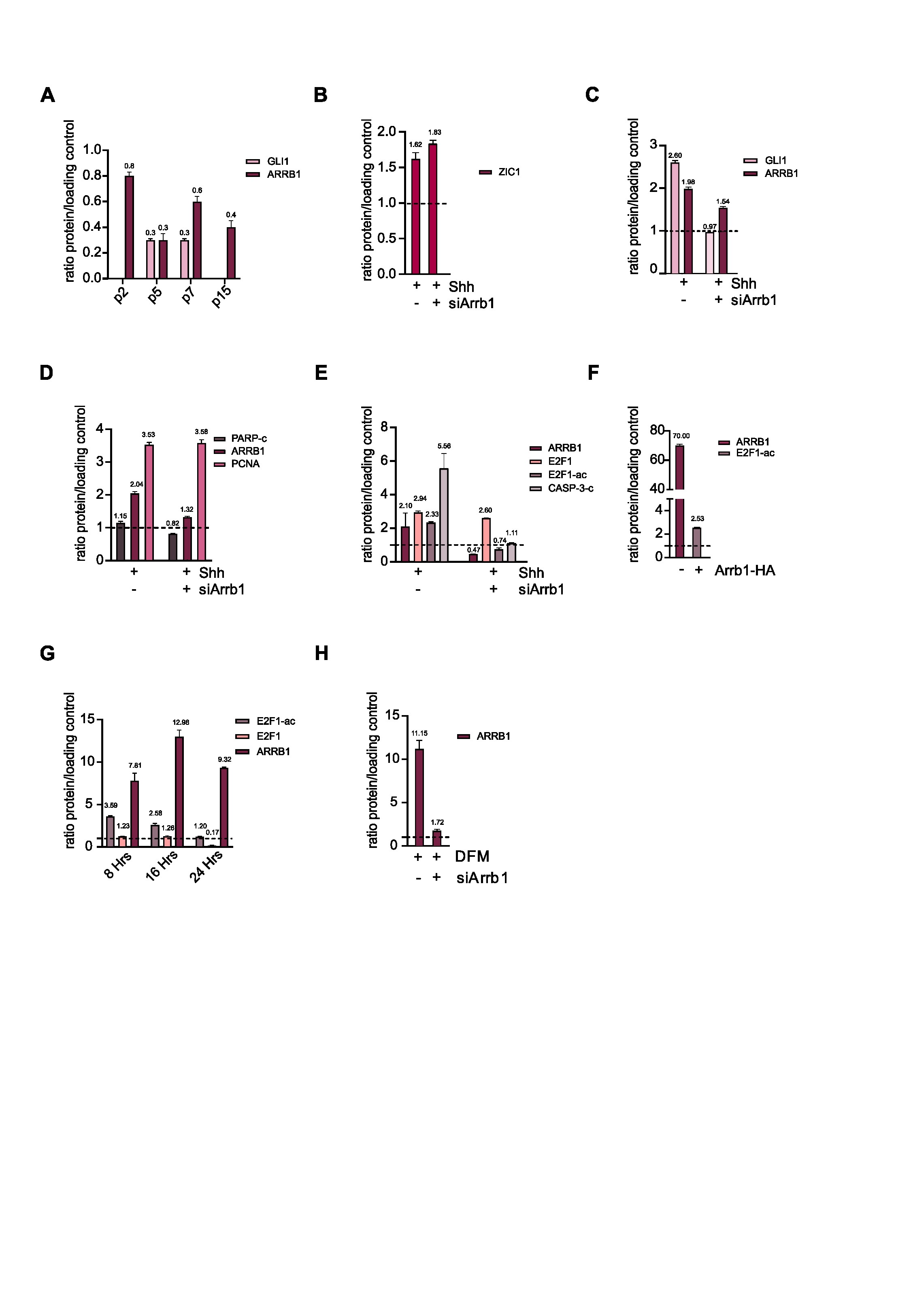

Supplement: Supplementary file 4 [file Image2.JPEG]
